# Real-world study of adverse events associated with gepant use in migraine treatment based on the VigiAccess and U.S. Food and Drug Administration’s adverse event reporting system databases

**DOI:** 10.3389/fphar.2024.1431562

**Published:** 2024-07-31

**Authors:** Qiaofang Liang, Xiaolin Liao, Hongwen Wu, Yushen Huang, Taolin Liang, Hailong Li

**Affiliations:** ^1^ Department of Pharmacy, The 4th Affiliated Hospital of Guangxi Medical University, Liuzhou, China; ^2^ Liuzhou Hospital of Traditional Chinese Medicine (Liuzhou Hospital of Zhuang Medicine), Liuzhou, China; ^3^ Department of Pharmacy, West China Second University Hospital, Sichuan University, Chengdu, China; ^4^ Evidence Based Pharmacy Center, West China Second University Hospital, Sichuan University, Chengdu, China; ^5^ Key Laboratory of Birth Defects and Related Diseases of Women and Children, Sichuan University, Ministry of Education, Chengdu, China

**Keywords:** gepant medications, adverse events, VigiAccess, food and drug administration adverse event reporting system, real-world, pharmacovigilance

## Abstract

**Background:**

This study aimed to investigate the real-world profile of adverse events (AEs) associated with gepant medications in the clinical treatment of migraines by analyzing data collected from the VigiAccess database and the U.S. Food and Drug Administration (FDA) Adverse Event Reporting System (FAERS) database. As novel migraine therapies, gepants act by targeting the calcitonin gene-related peptide (CGRP) pathway, demonstrating effective control of migraine attacks and good tolerability. Nonetheless, comprehensive real-world studies on the safety of gepants are still lacking, particularly regarding their safety in large populations, long-term use, and potential adverse reactions in specific groups, which necessitates further empirical research. Leveraging these two international adverse event reporting system databases, we systematically gathered and analyzed reports of AEs related to gepant medications, such as rimegepant. Our focus encompasses but is not limited to severe, new, and rare adverse reactions induced by the drugs, as well as safety issues pertaining to the gastrointestinal, cardiovascular, hepatic, and renal systems. Through descriptive statistical analyses, we assessed the incidence and characteristics of AEs, compared AEs among gepants, and uncovered previously unknown AE information, all with the goal of providing a reference for the selection of clinical treatment regimens and AE monitoring.

**Methods:**

By extracting all AE reports concerning “rimegepant”, “atogepant”, and “ubrogepant” from the VigiAccess and FAERS database since its establishment up to 31 March 2024, a retrospective quantitative analysis was conducted. The reporting odds ratio (ROR) method were used to compare AEs among the three gepants.

**Results:**

In the VigiAccess and FAERS databases, 23542 AE reports in total, respectively, were identified as being related to gepant medications. Among gastrointestinal system AEs, rimegepant had the greatest proportion and greatest signal strength; nausea was most severe and had the strongest signal in rimegepant AEs, whereas constipation was most prominent and had the strongest signal in atogepant AEs. In skin and subcutaneous tissue disorders, rash and pruritus were more frequently observed with rimegepant, followed by ubrogepant. Alopecia emerged as a novel AE, being more severe in rimegepant and secondarily in atogepant. Regarding cardiac disorders, the three gepants showed comparable rates of cardiac AEs, yet rimegepant exhibited the strongest AE signal. In musculoskeletal and connective tissue AEs, ubrogepant presented the most positive signals for skeletal muscle AEs. Furthermore, among the rare blood and lymphatic system disorder AEs, rimegepant had the highest number of reports of Raynaud’s phenomenon and the strongest signal. The study also revealed that while reports of AEs involving liver diseases were scarce across the three gepants, severe AEs were detected in clinical trials, highlighting the need for continued, enhanced monitoring of liver system AEs through large-scale datasets.

**Conclusion:**

Gepant medications exhibit similarities and differences in their safety profiles. Analysis of the two databases indicated the presence of AEs across various systems, including gastrointestinal disorders, skin and subcutaneous tissue diseases, musculoskeletal and connective tissue disorders, organ-specific effects, and liver diseases. However, each drug displays distinct incidences and signal intensities for these AEs. Additionally, the study revealed a rare AE in the form of Raynaud’s phenomenon. These findings suggest that during clinical use, individualized medication selection and AE monitoring should be based on the patient’s physiological condition and specific characteristics.

## Introduction

Migraine is a chronic and complex neurological disorder characterized by recurrent, predominantly unilateral, moderate-to-severe pulsating headaches, often accompanied by nausea and/or sensitivity to light and sound. According to the 2021 Global Burden of Disease Study, migraine ranks as the third leading cause of neurological health loss globally and is a major contributor to neurological disability (GBD, 2021 Nervous System Disorders Collaborators, 2024). Currently, several medications are used for the treatment of migraines, including antiepileptic drugs, antidepressants, analgesics, ergots, and triptans (Headache and Sensory Disorders Specialized Committee of the Chinese Society of Research Hospitals et al., 2022). However, for this particular neurological ailment, current medications are insufficient to meet the demands of clinical management (Dahlöf, 1993; Diener, 2020). Research has shown that gepant drugs, which target the calcitonin gene-related peptide (CGRP) pathway, consistently yield positive outcomes in migraine treatment, demonstrating greater efficacy and advantages than conventional migraine treatments. The American Headache Society recommends gepants when migraine control is inadequate or patients are intolerant to standard therapies (Charles and Rapoport, 2019) Currently available gepants include rimegepant, atogepant, ubrogepant, and zavegepant, with rimegepant approved for both acute and preventive treatment of migraines, ubrogepant for acute treatment, and atogepant used preventively. These drugs have lower lipophilicity and limited penetration of the blood‒brain barrier (Negro and Martelletti, 2021), and compared to triptans, they pose less risk of vasoconstrictive effects and medication-overuse headache (Durham, 2004; van Hoogstraten and MaassenVanDenBrink, 2019). Sharing similar mechanisms of action, gepants exhibit variations in the types and frequencies of adverse events (AEs) (Pozo-Rosich et al., 2023; Yu et al., 2023; Dodick et al., 2023). Most real-world studies to date support the safety, efficacy, and tolerability of gepants in migraine treatment (Dodick et al., 2019; Negro and Martelletti, 2020; Haanes and Edvinsson, 2023; Pozo-Rosich et al., 2023). However, post-marketing safety data are scarce, with much of the available information relying heavily on clinical trial results and meta-analyses based on these data (Haghdoost et al., 2023; Lee et al., 2022). Clinical trials, limited by sample size and follow-up duration, might underrepresent the incidence of less frequent or severe AEs, highlighting the need for comprehensive real-world studies.

The VigiAccess database serves as the World Health Organization (WHO) repository for collecting global adverse drug reaction data, while the Food and Drug Administration (FDA) Adverse Event Reporting System (FAERS) database encompasses detailed information on all medicines marketed in the United States, together encompassing a broad spectrum of drug users and representing crucial real-world data sources for AEs, thereby offering substantial data support for the early identification of AEs. Analyzing the AE data of patients receiving gepant medications using these two databases facilitates ongoing risk management for previously unrecognized safety signals, striving to mitigate the impact of AEs on patient treatment.

This study, through data mining on reports of gepant medications in the VigiAccess and FAERS databases, aims to provide in-depth insights into the safety of gepants for clinicians, patients, and regulatory bodies, guiding personalized treatment decisions and laying the groundwork for future pharmacovigilance activities and additional drug safety research. By synthesizing real-world evidence, we strive to comprehensively illustrate the safety-efficacy balance of gepants in migraine management, fostering safer and more efficacious migraine treatment.

## Materials and methods

### Data sources

The data for this study were sourced from the VigiAccess database, encompassing its entire history up until 31 March 2024, as well as the FAERS database, covering the period from Q1 2020 to Q4 2023. The focus was on all primary suspect AE reports pertaining to “rimegepant,” “atogepant,” and “ubrogepant.” The search was conducted using generic names, and AEs were described according to the System Organ Class (SOC) and Preferred Term (PT) of the Medical Dictionary for Regulatory Activities (MedDRA). The extracted data included patient demographics such as sex and age, report submission dates, geographic distribution, and the organ systems affected by the AEs. As of 31 March 2024, zavegepant had minimal entries in the VigiAccess database and no reported AEs in the FAERS database; consequently, this study did not undertake data mining and analysis for zavegepant.

### AE signal detection and statistical analysis methodology

A retrospective quantitative analysis was used to analyze all AE reports of the three gepant drugs in the VigiAccess database to understand the similarities, differences, characteristics and patterns of occurrence of AEs in the real world. The FAERS database AE signals were examined using the reporting odds ratio ( ROR , $\text{ROR}=\frac{\left(a/c\right)}{\left(b/d\right)}=\frac{ad}{bc}$ ; $95\%\text{CI}=e^{\ln\left(\text{ROR}\right)\pm 1.96\sqrt{\left(\frac{1}{a}+\frac{1}{b}+\frac{1}{c}+\frac{1}{d}\right)}}$ ). To avoid false-positive signals, positive AE signals should meet the lower limit of the 95% confidence interval (95% CI) of ROR > 1 and a ≥ 3, and these values were calculated based on the four-grid scale of the proportional imbalance measurement method (Table 1), The designated algorithm is implemented in accordance with (European Medicines Agency, 2008). Exclude AEs that are not related to the medication, ensuring that subsequent data analysis focuses on genuine drug-related AEs. The statistical analysis and graphing of the data were performed using SAS 9.4 software, Microsoft Office Excel 2019 and Python.

**TABLE 1:** Fourfold table of disproportionality measures.

	Target AE	OtherAE	Total
Target drugs	a	b	a+b
Other drugs	c	d	c + d
Total	a+c	b + d	a+b + c + d

## Results

### Basic information on the AE reports

By 31 March 2024, the following numbers of AE reports were gathered for the three medications from both the VigiAccess and FAERS databases: rimegepant with 6,949 and 6907reports, atogepant with 3,058 and 3,150 reports, and ubrogepant with 1759 and 1719 reports, respectively. With the exception of unknown reports, the ratio of females to males for all three drugs in both databases was roughly sixfold, exhibiting a broad range of variation. Furthermore, the predominant age groups reported fell between 45 and 64 years of age, and the Americas yielded the highest number of reports. The time span between 2021 and 2023 had the highest frequency of report submissions (Table 2).

**TABLE 2:** Demographic information reported by AE.

	Rimegepant				Atogepant				Ubrogepant			
	VigiAccess (n = 6,949)		FAERS (n = 6,907)		VigiAccess (n = 3,058)		FAERS (n = 3,150)		VigiAccess (n = 1759)		FAERS (n = 1719)	
	n	%	n	%	n	%	n	%	n	%	n	%
Sex												
Female	4,106	59.09	4,159	60.21	2,377	77.73	2,448	77.71	1,160	65.95	1,141	66.38
male	620	8.92	636	9.21	403	13.18	401	12.73	233	13.25	214	12.45
Unknown	2,223	31.99	2,112	30.58	278	9.09	301	9.56	376	21.38	364	21.18
Age (years)												
<18	46	0.66	42	0.61	3	0.10	4	0.13	37	2.10	34	1.98
18–44	1,023	14.72	1,048	15.17	302	9.88	301	9.56	140	7.96	132	7.68
45–64	1,405	20.22	1,468	21.25	419	13.70	432	13.71	180	10.23	175	10.18
≥65	560	8.06	565	8.18	91	2.98	101	3.21	66	3.75	69	4.01
Unknown	3,915	56.34	3,784	54.79	2,243	73.35	2,312	73.40	1,336	75.95	1,309	76.15
Reporting year												
2020	57	0.82	586	8.48	0	0.00	0	0.00	179	10.18	289	16.81
2021	1,535	22.09	1,680	24.32	1	0.03	18	0.57	465	26.44	350	20.36
2022	2,128	30.62	2,970	43.00	723	23.64	1,463	46.44	461	26.21	604	35.14
2023	2,967	42.70	1,394	20.18	2,203	72.04	1,518	48.19	583	33.14	392	22.80
2024	262	3.77	277	4.01	131	4.28	151	4.79	71	4.04	84	4.89
Geographical distribution												
Africa	1	0.01	0	0.00	0	0.00	0	0.00	0	0.00	0	0.00
Americas	6,868	98.83	6,866	99.41	3,055	99.90	3,146	99.87	1757	99.89	1705	99.19
Asia	11	0.16	9	0.13	0	0.00	2	0.06	2	0.11	3	0.17
Europe	69	0.99	30	0.43	3	0.10	2	0.06	0	0.00	0	0.00
Unknown	0	0.00	2	0.03	0	0.00	0	0.00	0	0.00	11	0.64

### AE report stratified analysis by SOC

When categorizing the AE signals of the three drugs according to SOC, general disorders and administration site conditions, nervous system disorders, and gastrointestinal disorders emerged as the top three SOC categories for the drugs in both the VigiAccess and FAERS databases. Specifically, within the FAERS database, atogepant and ubrogepant exhibited the strongest signals in the nervous system disorders category, whereas the most pronounced signal for rimegepant was related to general disorders and administration site conditions (Table 3).

**TABLE 3:** Distribution of AE signals in each SOC.

SOC	Rimegepant		Atogepant		Ubrogepant	
	VigiAccess n(%)	FAERS n (ROR)	VigiAccess n(%)	FAERS n (ROR)	VigiAccess n(%)	FAERS n (ROR)
General disorders and administration site conditions	5,294 (41.80)	5,220 (3.45)	1,306 (20.29)	1,336 (1.17)	1,108 (31.78)	1,026 (2.12)
Gastrointestinal disorders	1897 (14.98)	1853 (1.03)	1,009 (15.68)	1,069 (2.18)	419 (12.02)	379 (1.49)
Nervous system disorders	1,637 (12.92)	1,560 (1.79)	1,683 (26.15)	1706 (4.21)	789 (22.63)	747 (3.62)
Injury, poisoning and procedural complications	850 (6.71)	845 (0.54)	446 (6.93)	454 (0.53)	294 (8.43)	303 (0.74)
Skin and subcutaneous tissue disorders	569 (4.49)	571 (0.77)	186 (2.89)	201 (0.49)	101 (2.90)	97 (0.48)
Psychiatric disorders	480 (3.79)	438 (0.65)	312 (4.85)	343 (0.95)	135 (3.87)	120 (0.67)
lmmune system disorders	248 (1.96)	239 (1.60)	47 (0.73)	50 (0.61)	35 (1.00)	28 (0.69)
Respiratory, thoracic and mediastinal disorders	246 (1.94)	238 (0.42)	118 (1.83)	123 (0.39)	66 (1.89)	64 (0.42)
Product issues	182 (1.44)	183 (0.82)	8 (0.12)	7 (0.06)	18 (0.52)	21 (0.35)
Investigations	188 (1.48)	184 (0.25)	180 (2.8)	203 (0.51)	74 (2.12)	63 (0.32)
Musculoskeletal and connective tissue disorders	213 (1.68)	196 (0.31)	146 (2.27)	156 (0.45)	92 (2.64)	88 (0.52)
Eye disorders	159 (1.26)	151 (0.64)	121 (1.88)	117 (0.91)	61 (1.75)	74 (1.18)
Infections and infestations	149 (1.18)	140 (0.20)	163 (2.53)	177 (0.46)	47 (1.35)	47 (0.25)
Ear and labyrinth disorders	104 (0.82)	93 (1.81)	61 (0.95)	62 (2.22)	32 (0.92)	30 (2.19)
Vascular disorders	99 (0.78)	95 (0.41)	53 (0.82)	56 (0.44)	36 (1.03)	30 (0.48)
Cardiac disorders	81 (0.64)	78 (0.31)	51 (0.79)	51 (0.37)	29 (0.83)	31 (0.46)
Metabolism and nutrition disorders	56 (0.44)	48 (0.19)	115 (1.79)	124 (0.94)	23 (0.66)	16 (0.24)
Surgical and medical procedures	35 (0.28)	34 (0.19)	272 (4.23)	278 (3.01)	52 (1.49)	46 (0.98)
Reproductive system and breast disorders	36 (0.28)	36 (0.44)	38 (0.59)	42 (0.94)	12 (0.34)	11 (0.5)
Renal and urinary disorders	33 (0.26)	35 (0.13)	25 (0.39)	29 (0.21)	13 (0.37)	12 (0.17)
Social circumstances	30 (0.24)	27 (0.46)	25 (0.39)	30 (0.95)	19 (0.55)	18 (1.16)
Neoplasms benign, malignant and unspecified (incl cysts and polyps)	22 (0.17)	17 (0.04)	36 (0.56)	46 (0.19)	9 (0.26)	9 (0.08)
Pregnancy, puerperium and perinatal conditions	20 (0.16)	10 (0.22)	13 (0.20)	13 (0.52)	7 (0.20)	20 (1.62)
Hepatobiliary disorders	18 (0.14)	16 (0.15)	7 (0.11)	15 (0.27)	3 (0.09)	3 (0.11)
Congenital, familial and genetic disorders	7 (0.06)	6 (0.19)	5 (0.08)	5 (0.28)	2 (0.06)	2 (0.23)
Endocrine disorders	6 (0.05)	4 (0.12)	6 (0.09)	8 (0.45)	5 (0.14)	2 (0.23)
Blood and lymphatic system disorders	7 (0.06)	4 (0.02)	4 (0.06)	5 (0.04)	5 (0.14)	7 (0.13)

### Analysis of AE signals at the PT level

Next, all signals at the PT level were analyzed, focusing on the top 30 most frequent and highest signal strength detections appearing in both the VigiAccess and FAERS databases. In the VigiAccess database, among gastrointestinal disorders, nausea emerged as the most severe AE for rimegepant, while constipation was most prevalent for atogepant, followed by rimegepant. Among skin and subcutaneous tissue disorders, rimegepant showed higher frequencies of rash and pruritus. Alopecia seemed more common with rimegepant than with atogepant. Among cardiac disorders and musculoskeletal and connective tissue disorders, ubrogepant accounted for a large proportion of the detected AEs (Table 4.) In the FAERS database, the pattern of nausea and constipation predominance among gastrointestinal system signals aligns with the VigiAccess findings, maintaining their respective signal strengths. Strong signals for cardiac system AEs, such as cardiac flutter, were observed with atogepant. Musculoskeletal AEs such as muscle tightness and neck pain showed an increased number of signals for ubrogepant. Among vascular disorders, Raynaud’s phenomenon signals were stronger for rimegepant, followed by atogepant. No positive signals were detected for hepatobiliary disorders (Table 5).

**TABLE 4:** Top 30 AEs with the highest percentage of signal detection in the VigiAccess database.

NO	Rimegepant		Atogepant		Ubrogepant	
	PT	n(%)	PT	n(%)	PT	n(%)
1	Drug ineffective	2,906 (22.94)	Migraine	696 (10.81)	Drug ineffective	583 (16.72)
2	Nausea	717 (5.66)	Drug ineffective	481 (7.47)	Nausea	163 (4.68)
3	Migraine	330 (2.61)	Headache	405 (6.29)	Off label use	103 (2.95)
4	Headache	329 (2.60)	Nausea	350 (5.44)	Fatigue	61 (1.75)
5	Dizziness	235 (1.86)	Constipation	262 (4.07)	Vomiting	54 (1.55)
6	Off label use	212 (1.67)	Fatigue	212 (3.29)	Feeling abnormal	44 (1.26)
7	Feeling abnormal	187 (1.48)	Dizziness	128 (1.99)	Anxiety	38 (1.09)
8	Vomiting	184 (1.45)	Vomiting	76 (1.18)	Pain	29 (0.83)
9	Somnolence	159 (1.26)	Somnolence	75 (1.17)	Insomnia	26 (0.75)
10	Abdominal pain upper	156 (1.23)	Off label use	74 (1.15)	Constipation	26 (0.75)
11	Fatigue	144 (1.14)	Decreased appetite	73 (1.13)	Diarrhoea	26 (0.75)
12	Rash	142 (1.12)	Feeling abnormal	71 (1.10)	Chest pain	26 (0.75)
13	Hypersensitivity	141 (1.11)	Weight decreased	54 (0.84)	Abdominal discomfort	23 (0.66)
14	Abdominal discomfort	121 (0.96)	Insomnia	51 (0.79)	Abdominal pain upper	23 (0.66)
15	Pruritus	108 (0.85)	Malaise	50 (0.78)	Rash	23 (0.66)
16	Dyspepsia	91 (0.72)	Anxiety	49 (0.76)	Malaise	21 (0.60)
17	Pain	87 (0.69)	Alopecia	48 (0.75)	Pruritus	20 (0.57)
18	Constipation	83 (0.66)	Pain	45 (0.70)	Anxiety	19 (0.55)
19	Malaise	83 (0.66)	Abdominal pain upper	43 (0.67)	Dyspnoea	18 (0.52)
20	Diarrhoea	78 (0.62)	Pruritus	39 (0.61)	Palpitations	16 (0.46)
21	Anxiety	78 (0.62)	Diarrhoea	37 (0.57)	Vertigo	16 (0.46)
22	Dyspnoea	78 (0.62)	Gastrointestinal disorder	32 (0.50)	Chest discomfort	14 (0.40)
23	Urticaria	74 (0.58)	Vertigo	31 (0.48)	Death	13 (0.37)
24	Unevaluable event	70 (0.55)	Rash	31 (0.48)	Insomnia	13 (0.37)
25	Insomnia	69 (0.54)	Adverse event	30 (0.47)	Hypersensitivity	12 (0.34)
26	illness	66 (0.52)	Depression	28 (0.44)	Urticaria	12 (0.34)
27	Paraesthesia	57 (0.45)	Hypersensitivity	27 (0.42)	Hypertension	12 (0.34)
28	Drug hypersensitivity	54 (0.43)	Fall	27 (0.42)	Chills	11 (0.32)
29	Alopecia	51 (0.40)	Seizure	27 (0.42)	Drug hypersensitivity	11 (0.32)
30	Gastrointestinal disorder	49 (0.39)	Abdominal discomfort	26 (0.40)	Muscle spasms	11 (0.32)

**TABLE 5:** Top 30 AEs with the highest signal intensity in the FAERS database AE signal detection.

	Rimegepant			Atogepant			Ubrogepant		
	PT	a	ROR (95%CI lower)	PT	a	ROR (95%CI lower)	PT	a	ROR (95%CI lower)
1	Medication overuse headache	22	113.81 (74.12)	Post concussion syndrome	3	88.78 (28.27)	Habitual abortion	13	5,982.5 (2,928.89)
2	Aura	14	45.72 (26.92)	Migraine	695	74.91 (69.21)	Medication overuse headache	8	150.04 (74.50)
3	Hangover	11	18.35 (10.13)	Cluster headache	7	46.25 (21.95)	Aura	7	84.74 (40.21)
4	Migraine with aura	9	17.18 (8.91)	Premenstrual syndrome	3	32.43 (10.41)	Autoscopy	3	65.2 (20.92)
5	Migraine	311	16.62 (14.85)	Migraine with aura	6	21 (9.41)	Migraine	231	48.33 (42.27)
6	Morning sickness	3	16.5 (5.30)	Aura	3	17.7 (5.69)	Cluster headache	3	40.09 (12.89)
7	Nasal oedema	4	15.06 (5.63)	Therapy interrupted	169	16.77 (14.39)	Migraine with aura	3	21.32 (6.86)
8	Tension headache	14	14.53 (8.59)	Hyperacusis	6	16.69 (7.48)	Hyperacusis	3	16.96 (5.46)
9	Drug ineffective	2,890	12.83 (12.30)	Motion sickness	3	16.46 (5.29)	Feeling drunk	4	13.8 (5.17)
10	Performance status decreased	9	12.7 (6.59)	Brain fog	16	15.5 (9.48)	Tension headache	3	11.59 (3.73)
11	Motion sickness	4	11.96 (4.47)	Constipation	277	12.61 (11.18)	Photopsia	3	11.43 (3.68)
12	Inhibitory drug interaction	3	11.29 (3.63)	Self-injurious ideation	3	8.16 (2.63)	Euphoric mood	5	11.2 (4.65)
13	Tongue discomfort	12	9.78 (5.55)	Abnormal dreams	13	7.74 (4.49)	Paraesthesia oral	7	10.08 (4.80)
14	Electric shock sensation	6	8.87 (3.98)	Tension headache	4	7.59 (2.84)	Drug ineffective	544	8.25 (7.53)
15	Feeling drunk	9	8.31 (4.32)	Impaired gastric emptying	6	6.71 (3.01)	Head discomfort	8	7.72 (3.85)
16	Paraesthesia oral	20	7.71 (4.97)	Headache	403	6.65 (6.02)	Photophobia	7	7.65 (3.64)
17	Hyperacusis	5	7.56 (3.14)	Paradoxical drug reaction	3	6.62 (2.13)	Hypoaesthesia oral	4	6 (2.25)
18	Hypoaesthesia oral	18	7.23 (4.55)	Photophobia	12	6.44 (3.65)	Somnolence	58	5.97 (4.60)
19	Head discomfort	27	6.97 (4.78)	Head discomfort	13	6.16 (3.57)	Vertigo	15	5.12 (3.08)
20	Raynaud’s phenomenon	6	6.51 (2.92)	Raynaud’s phenomenon	3	5.98 (1.93)	Abnormal dreams	4	4.84 (1.82)
21	Vomiting projectile	3	6.31 (2.03)	Euphoric mood	5	5.49 (2.28)	Throat tightness	6	4.79 (2.15)
22	Dyspepsia	89	5.33 (4.32)	Vertigo	31	5.2 (3.66)	Feeling cold	6	4.48 (2.01)
23	Sinus headache	4	5.3 (1.99)	Gastrointestinal motility disorder	3	5.16 (1.66)	Nausea	151	4.08 (3.46)
24	Ear pruritus	4	5.27 (1.98)	Food poisoning	3	5 (1.61)	Muscle tightness	3	4.06 (1.31)
25	Pharyngeal swelling	16	5.17 (3.16)	Paraesthesia oral	7	4.95 (2.36)	Headache	123	4.03 (3.37)
26	Nausea	694	5.07 (4.70)	Nausea	358	4.79 (4.31)	Therapeutic response unexpected	9	4 (2.08)
27	Unevaluable event	67	4.95 (3.89)	Cardiac flutter	3	4.69 (1.51)	Neck pain	11	3.97 (2.20)
28	Drug tolerance	5	4.9 (2.04)	Tongue discomfort	3	4.48 (1.44)	Therapy interrupted	20	3.95 (2.54)
29	Therapeutic response unexpected	38	4.52 (3.29)	Amenorrhoea	5	4.2 (1.75)	Head injury	6	3.93 (1.77)
30	Swollen tongue	22	4.45 (2.93)	Parosmia	3	4.19 (1.35)	Cold sweat	3	3.88 (1.25)

### Comparison of AE signals

**FIGURE 1:**
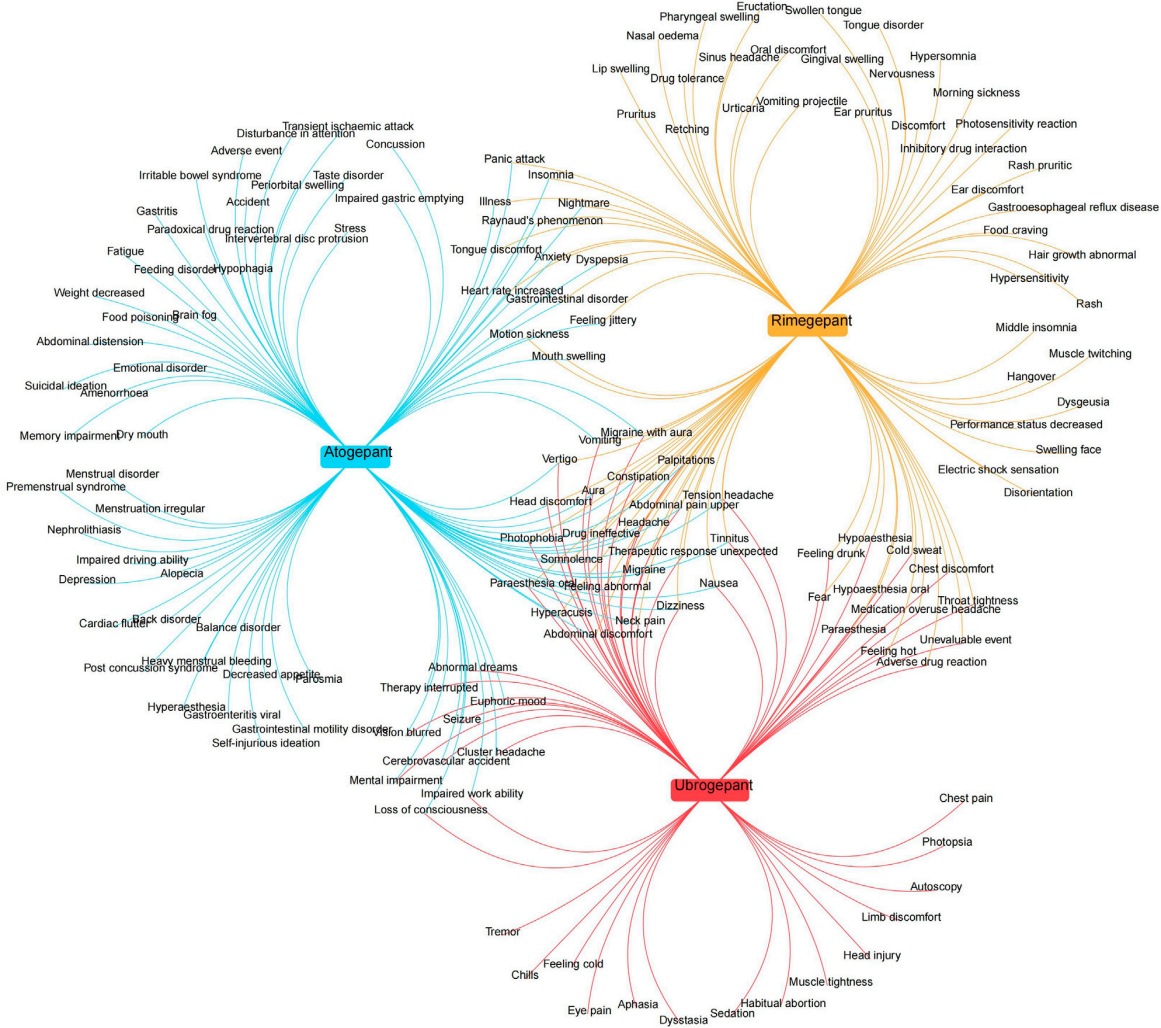
Network Venn diagrams of PT-positive signals in the FAERS database for the three gepant drugs.

A comparative analysis of the AE signals identified for the three gepant medications was conducted. According to the FAERS database, rimegepant exhibited the greatest number of positive AE signals among the three gepants. A total of 24 AE signals were common to all three drugs. (Figure 1). This comparison highlights both the overlapping nature of certain AEs across these medications and the unique profiles each drug presents, contributing to a nuanced understanding of their safety profiles in clinical use.

## Discussion

The introduction of gepant medications has expanded the spectrum of options for migraine treatment, offering more alternatives in therapeutic decision-making. Beyond evaluating their efficacy, ensuring safety remains a pivotal concern when are assessed these drugs. Investigating the real-world characteristics of AEs associated with gepants contributes to a more comprehensive and precise clinical judgment in drug selection. Analysis revealed that AEs related to gepants exhibit broad-ranging manifestations in actual clinical practice, implicating multiple organ systems. By comparing and analyzing the AEs of rimegepant, atogepant, and ubrogepant reported in the VigiAccess and FAERS databases, this study provides a thorough understanding of the similarities and differences in the safety profiles of these three drugs across common, novel, and rare AEs. All AEs listed in the medication package inserts were identified through our data mining process. Furthermore, common AEs in this research displayed high incidences and signal intensities, reinforcing the importance of considering their prevalence and impact in clinical settings.

AEs related to the gastrointestinal system were observed with all three gepants. Mechanisti-cally, CGRP, which is widely distributed in interstitial neurons of the gastrointestinal tract and facilitates increased intestinal blood flow and smooth muscle relaxation, is antagonized by gepants. These drugs affect not only CGRP receptors in the central nervous system but also those dominant in the enteric nervous system. Animal studies have demonstrated a dose-dependent effect of CGRP blockade on motility, suppressing CGRP release from gastrointestinal neurons and thereby contributing to gastrointestinal AEs (Yang et al., 2022). However, there are variations in the incidence of these AEs among the three gepants, with atogepant showing the highest frequency, followed by rimegepant, consistent with the order of signal strength revealed in the FAERS database. For high-risk patients requiring continued prophylactic treatment with gepants, rimegepant might be considered an alternative option. Notably, rimegepant exhibits a notably high rate and signal intensity of AEs in the oral cavity, which can negatively impact patients’ quality of life and adherence to therapy, warranting attention in clinical practice (Figure 1).

In the context of skin and subcutaneous tissue disorders, manifestations include various forms, such as rash, pruritus, and alopecia. CGRP, a crucial neuromodulator in wound healing, also regulates the immune status of the skin (Lu et al., 2024). CGRP-positive fibers in the epidermis interact directly with Langerhans cells, reducing their antigen-presenting capacity and promoting immune tolerance. The anti-inflammatory effect of CGRP inhibition on other immune cells counteracts this immunosuppressive effect. Simultaneously, CGRP stimulates keratinocyte proliferation but also prompts the release of CGRP, interleukin-1β (IL-1β), IL-6, and tumor necrosis factor-alpha (TNF-α), leading to conditions such as rash and pruritus (Shi et al., 2013). The mechanism underlying alopecia, a newly reported AE associated with gepants, caused by these drugs is unclear; however, several studies support a potential link between CGRP receptor antagonists and hair loss, possibly involving microvascular circulation disruption and other homeostatic mechanisms. Existing evidence consistently indicates that hair loss signals are associated with CGRP inhibitors (Rossi et al., 1997; Harada et al., 2007; Woods, 2022; Ruiz et al., 2023). Therefore, when clinically administering gepants, collaboration with dermatologists for specialized treatment or close monitoring is advisable, and further pharmacovigilance research in this area is warranted.

In terms of cardiac disorders, although clinical trials of gepants have suggested that the class is safe and effective in patients with cardiovascular risk factors, all pivotal trials excluded patients with myocardial infarction, acute coronary syndrome, percutaneous coronary intervention, cardiac surgery, stroke or transient is chaemic attack within 6 months of screening (Powell et al., 2023). Nerve fibres releasing CGRP, a potent vasodilatory peptide, are widely distributed throughout the cardiovascular system and CGRP is essential for the regulation of cardiovascular function and protection of the myocardium (Jiarong and Dajiang, 2017). As a CGRP receptor antagonist, gepant has shown efficacy in the treatment of migraine, for example, while reducing the risk of cardiovascular side effects of traptans, making it more suitable for patients with a history of cardiovascular disease. However, cardiac AEs were seen with the gepant analogues in data from both systems, with atogepant in particular showing a stronger AE signal (Figure 1). This may attribute to the fact that a patient’s cardiovascular risk or disease base may increase the likelihood of a cardiac AE; post-marketing AE monitoring systems may be affected by reporting bias, i.e., serious or rare AEs are more likely to be reported, whereas minor or temporary AEs may be under-reported, leading to over- or underestimation of the incidence of cardiac AEs; the new AEs including cardiovascular AEs may emerge after prolonged and widespread use; polypharmacy may increase the risk of cardiac AEs. This finding suggests that we may need to reassess the safety of this class of drugs in patients with cardiovascular disease. Therefore, continued monitoring and further studies are essential to clarify the cardiovascular safety of these drugs when used in patients with cardiovascular disease.

Furthermore, our analysis revealed musculoskeletal-related AEs associated with gepant medications. A possible mechanism involves CGRP, which is abundant in the periosteum and bone marrow and plays a role in promoting the expression of osteogenic mediators during fracture healing. Studies have shown that the activation of CGRP receptor-mediated signaling facilitates tendon-bone healing in mice and is crucial for skin and muscle regeneration processes (Zhao et al., 2024). As CGRP receptor antagonists, gepants disrupt the normal physiological activities of CGRP, leading to musculoskeletal AEs. Under the classification of musculoskeletal and connective tissue disorders, skeletal muscle AEs related to gepants were identified, with ubrogepant exhibiting the greatest number of positive signals for such AEs (Figure 1). Consequently, for acute migraine patients presenting with bone, joint, or muscular symptoms, rimegepant might be a more favorable choice given its relative profile.

The study also identified Raynaud’s phenomenon as a rare AE signal associated with gepant-class drugs, with case reports and studies already documenting this AE (Bedrin et al., 2022; Singh et al., 2024). Episodic, symmetric distal limb (primarily fingers) pallor, cyanosis, and erythema significantly impair patients’ quality of life and safety. This impairment highlights the need in clinical practice not only to monitor for common AEs but also to remain vigilant for rare AEs, ensuring timely intervention.

Hepatobiliary disorder AEs are key signals of concern in the development of gepants, with potential hepatotoxicity being a primary concern in preventive clinical trials. Currently marketed gepants have significantly improved liver toxicity profiles; however, in clinical trials, alanine aminotransferase (ALT) or aspartate aminotransferase (AST) levels exceeded three times the upper limit of normal during atogepant treatment in four patients (Tassorelli et al., 2024). Given the potentially fatal consequences of severe hepatotoxicity, close monitoring of liver function parameters and signs is clinically warranted for patients receiving gepant therapy.

The safety and efficacy of gepants for the treatment of migraine depend not only on the features of the drug itself, but also on the specific situation of the patient, polypharmacy, and drug-drug interactions. Treatment strategies for migraine often involve multimodal therapy, including prophylactic and acute treatment, which may involve combining drugs with different mechanisms, such as a combination with CGRP monoclonal antibodies. Such combined therapies aim to relieve pain and reduce the frequency of attacks through different means, but also increase the risk of drug-drug interactions. Secondly, the interaction of drugs with the enzyme CYP3A4 is an important consideration. CYP3A4 inhibitors slow down the metabolism of gepants and increase their levels in the blood, potentially worsening side effects; conversely, CYP3A4 inducers can accelerate their metabolism and reduce their efficacy. At the same time, P-gp inhibitors may also affect the absorption and distribution of the drug, further affecting its efficacy and safety. Despite the potential for interactions, it has been shown that gepants can be added as adjunctive therapy during prophylactic CGRP monotherapy, that co-administration does not affect pharmacokinetics or safety, and that it does not increase the incidence of AEs, providing important guidance to clinical practice (Berman et al., 2020; Mullin et al., 2020; Jakate et al., 2021). In light of these complexities, clinicians prescribing gepants should inquire about the patient’s medication history in detail, particularly whether they are co-using CYP3A4 inhibitors/inducers or P-gp inhibitors, and adjust the dose or choose an alternative prescription if necessary. At the same time, monitor the patient’s response and any possible AEs, and adjust the treatment plan in a timely manner to ensure safety and efficacy.

This study has several inherent limitations. The research data are derived from a spontaneous reporting database, which is known to be subject to biases, including underreporting, duplication, stimulated reporting, and other factors that may confound the analysis. The quality and completeness of the data are also less than optimal. Nonetheless, the findings of this large-scale data mining serve as a warning for the safe and rational use of medications. All signal detection outcomes can only indicate statistical associations; they do not establish prevalence or confirm causal relationships. Furthermore, signal strength merely represents the relative magnitude of risk and does not quantify absolute risk. It is generally challenging to manage AEs of preventive migraine medications, especially given the history of numerous failed prior preventive treatments. Future studies should build upon the AE signals identified herein and conduct further high-quality research to clarify the incidence of these AEs in real-world usage scenarios.

Zavegepant, which was introduced to the market later, is not yet recorded in the FAERS database, and only limited data are available in the VigiAccess database; hence, it was not included in this study. Zavegepant, a third-generation small-molecule CGRP receptor antagonist, stands as the sole gepant in clinical development with a nasal formulation. According to published results from phase III clinical trials, zavegepant demonstrates good tolerability, with the most frequent AEs being taste disturbances, nausea, nasal discomfort, and vomiting. Most AEs are mild or moderate in severity and resolve without intervention, and no hepatotoxicity signals have been observed (Croop et al., 2022; Lipton et al., 2023). Given its recent market entry compared to that of other gepants, extensive real-world AE data for zavegepant are currently lacking. In subsequent research, investigators will need to encompass populations using zavegepant, facilitating the discovery of previously unknown AE signals associated with zavegepant through real-world studies. Doing so will enable a more comprehensive understanding of the AE profile of zavegepant, aiding in the clinical management and surveillance of AEs and the selection of treatment regimens.

The current mechanism of gepants in the treatment of migraine is based on the antagonism of the CGRP receptor, which plays a key role in the pathophysiology of migraine and whose levels are elevated during migraine attacks. CGRP receptor antagonists can effectively relieve migraine. The CGRP receptor we are referring to is actually the CGRP1 receptor (CLR/RAMP1), which is currently classified as a ‘CGRP receptor’. It has been shown that there is another receptor for CGRP, the AMY1 receptor (CTR/RAMP1), which belongs to the second trigeminal CGRP receptor and is present at important sites of craniofacial pain. The AMY1 receptor (CTR/RAMP1) shares RAMP1 as a co-protein with the CGRP1 receptor (CLR/RAMP1), and certain drugs that target at the CGRP pathway act not only on CGRP, but also on RAMP1(Walker et al., 2015). The specific function of the AMY1 receptor, particularly its role in the pathogenesis of migraine, is not fully understood. However, some studies have suggested that it may be involved in pain transmission or other physiological processes. A report by Ghanizada et al. (2021) proposed that pancreatic amylase analogue-triggered migraine-like headache is associated with the AMY1 receptor and that pancreatic amyloid polypeptide receptor agonism is a novel factor in the pathogenesis of migraine. Therefore, the researchers suggest that greater therapeutic efficacy in migraine patients may be achieved by dual antagonism of pancreatic amyloid polypeptide and CGRP receptors, rather than selective targeting of the typical CGRP receptor. Future research directions should include detailed characterization of the specific mechanisms of action of CGRP and AMY1 receptors in migraine and other related pain conditions, as well as the development of more specific drugs to minimize potential side effects while maximizing therapeutic benefits. As more data accumulated, we expect to gain a clearer understanding of the function of these receptors and how to target them more precisely to optimize therapeutic strategies for migraine.

## Data Availability

The datasets presented in this study can be found in online repositories. The names of the repository/repositories and accession number(s) can be found below: https://www.vigiaccess.org/; https://www.fda.gov/drugs/questions-and-answers-fdas-adverse-event-reporting-system-faers/fda-adverse-event-reporting-system-faers-public-dashboard.
